# Supplementary figures and images for: Investigating the accuracy of imputing autosomal variants in Nellore cattle using the ARS-UCD1.2 assembly of the bovine genome
Source: BMC Genomics. 2020 Nov 10;21:772. doi: 10.1186/s12864-020-07184-8 (PMC7654006; doi:10.1186/s12864-020-07184-8)

a

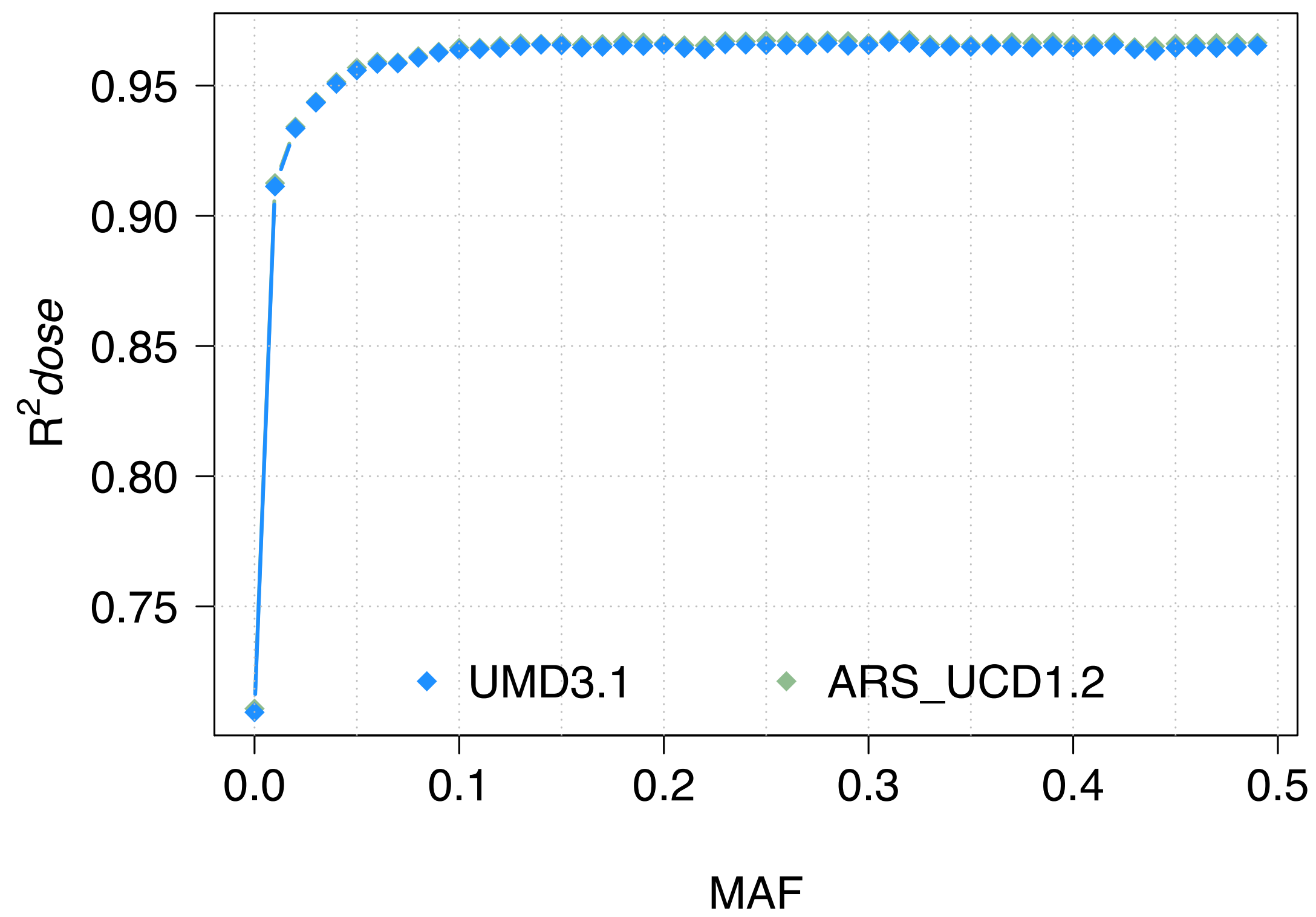

b

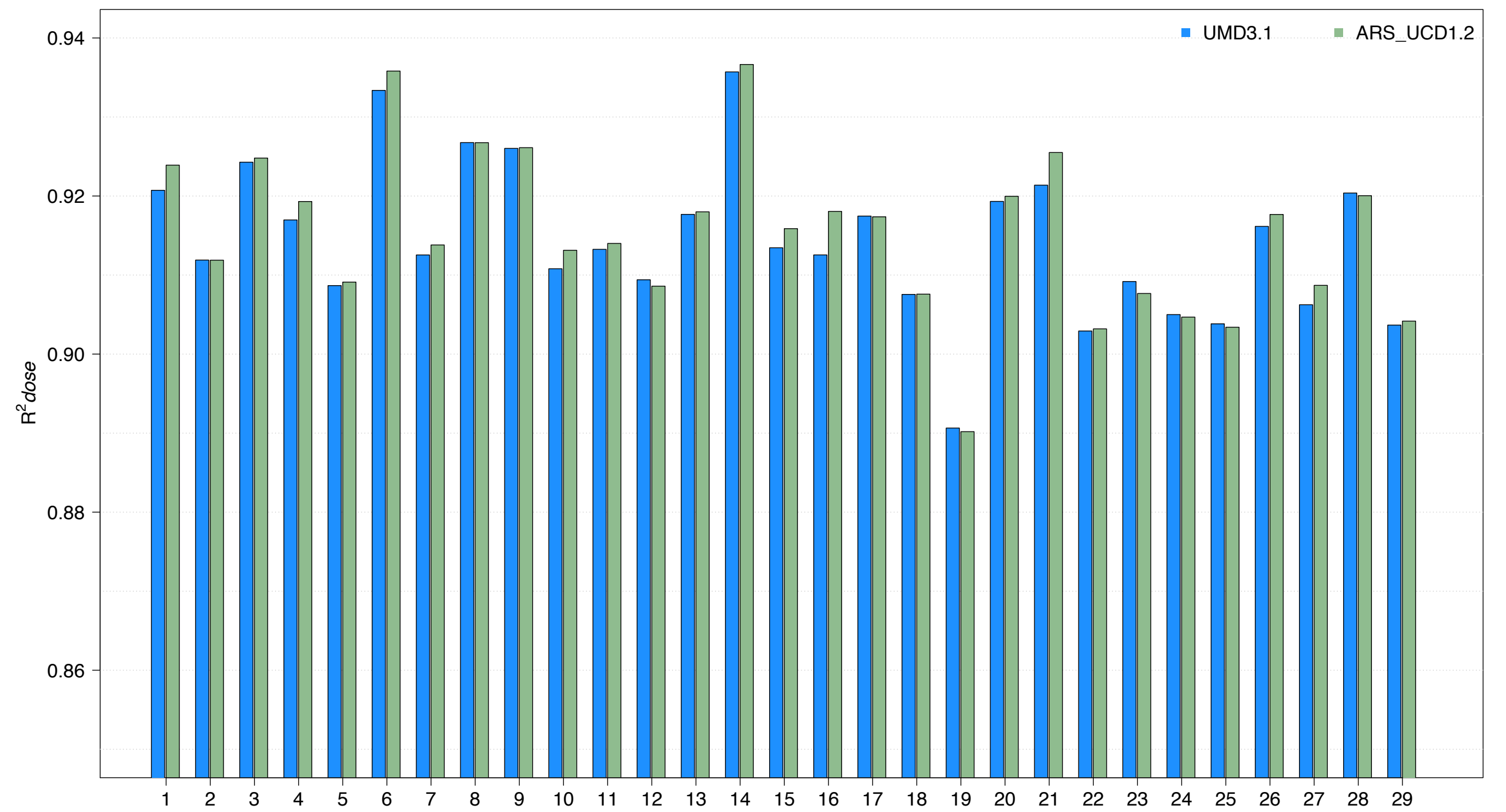

Supplement: Supplementary file 1 — Additional file 1. Imputation accuracy according to reference genome assembly. a) Accuracy of imputation for markers grouped in MAF bins when aligned to the two Bovine reference assemblies (ARS-UCD1.2 and UMD3.1). b) The mean imputation accuracy for markers on the 29 autosomes when aligned to the two Bovine assemblies. [file 12864_2020_7184_MOESM1_ESM.pdf]

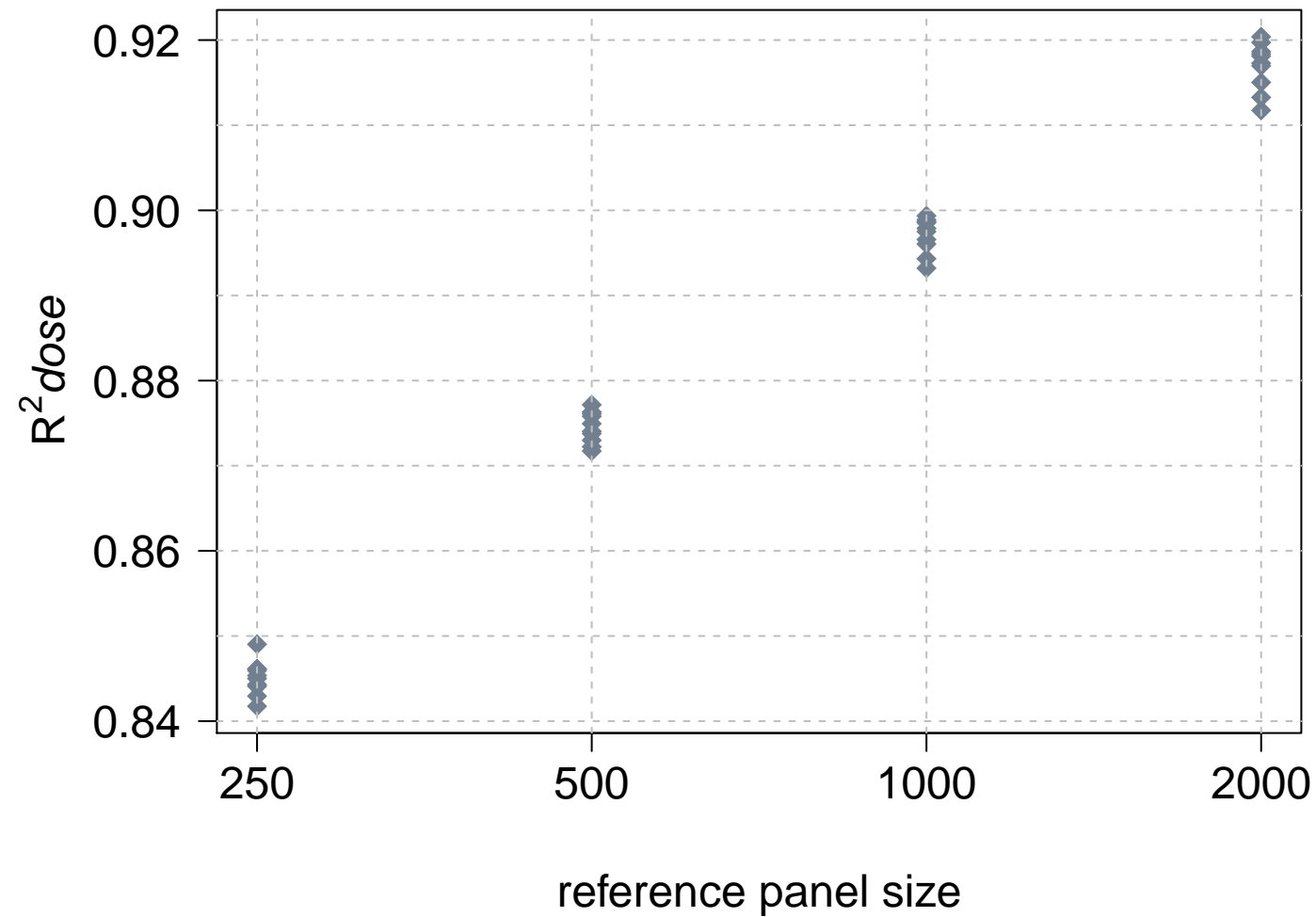

Supplement: Supplementary file 4 — Additional file 4. Variance in R2dose across 10 replicates. The variance in imputation accuracy (R2dose) across 10 replicates when imputing reference panels of different sizes. [file 12864_2020_7184_MOESM4_ESM.pdf]

Imputation accuracy

N= 250

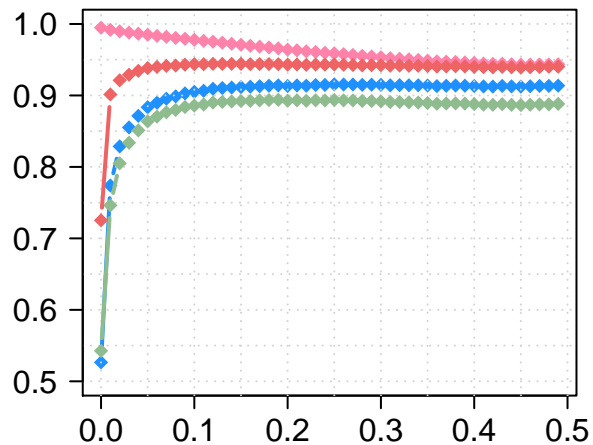

N= 500

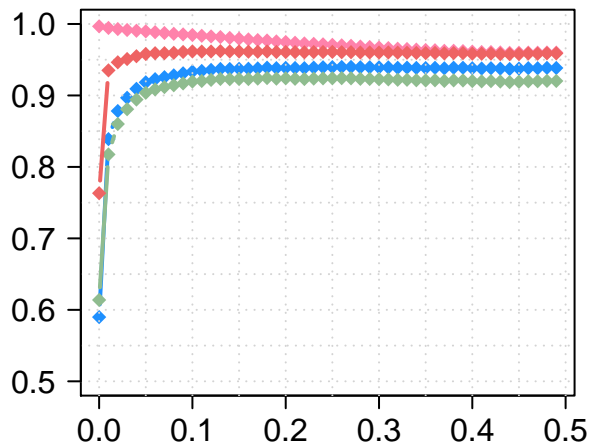

N= 1000

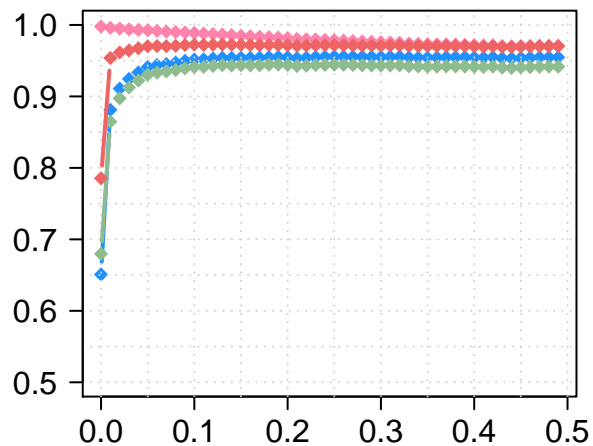

N= 2000

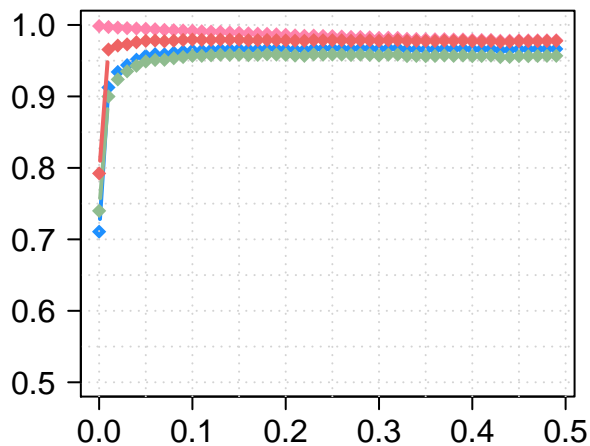

$R^2_{dose}$   
 $R^2_{gt}$   
CR  
 $Rsq$

MAF

Supplement: Supplementary file 5 — Additional file 5. Model-based imputation quality score from Minimac3 (Rsq) and empirical measures of imputation accuracy. Empirical measures of accuracy and the model-based imputation quality scores from Minimac3 (Rsq) for markers grouped in MAF bins when imputing with reference panels of varying sizes. [file 12864_2020_7184_MOESM5_ESM.pdf]

Rsq - Empirical Accuracy

N= 250

N= 500

N= 1000

N= 2000

$R^2_{dose}$   
 $R^2_{gt}$   
CR

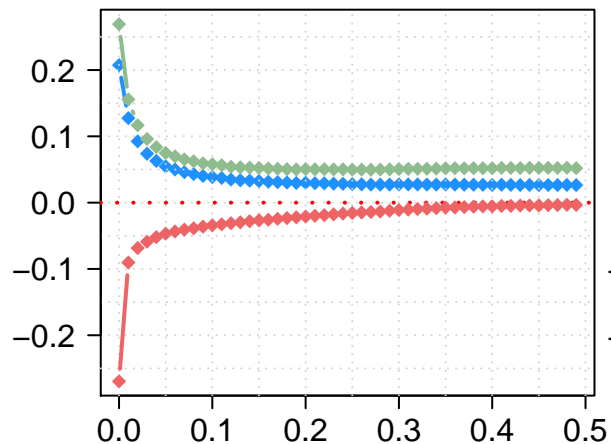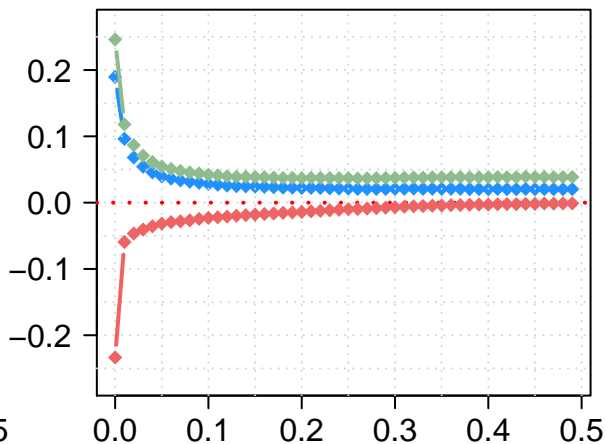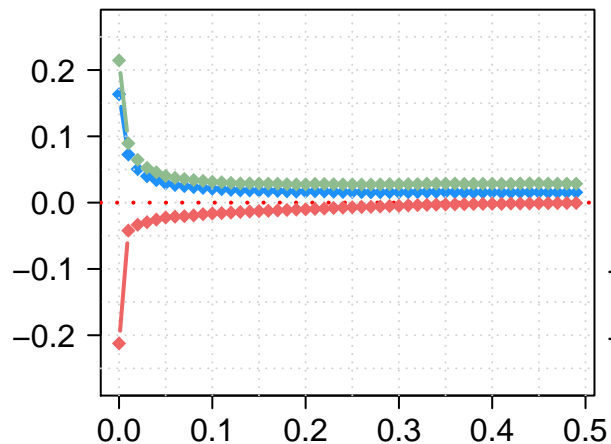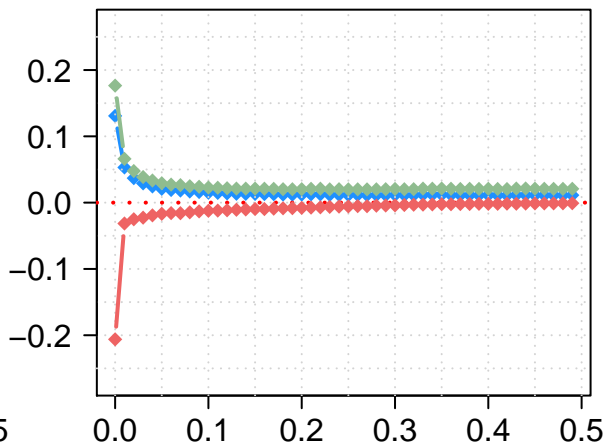

MAF

Supplement: Supplementary file 6 — Additional file 6. Discrepancy between the model-based imputation quality scores from Minimac3 (Rsq) and empirical measures of accuracy. Discrepancy between the model-based imputation quality scores from Minimac3 (Rsq) and empirical measures of accuracy for markers grouped in MAF bins when imputing with reference panels of varying sizes. [file 12864_2020_7184_MOESM6_ESM.pdf]
